# Deep Learning-Based Acoustic Emission Scheme for Nondestructive Localization of Cracks in Train Rails under a Load

**DOI:** 10.3390/s21010272

**Published:** 2021-01-03

**Authors:** Wara Suwansin, Pattarapong Phasukkit

**Affiliations:** School of Engineering, King Mongkut’s Institute of Technology Ladkrabang, Bangkok 10520, Thailand; 58601023@kmitl.ac.th

**Keywords:** acoustic emission sensor, acoustic emission testing, deep learning, nondestructive testing (NDT)

## Abstract

This research proposes a nondestructive single-sensor acoustic emission (AE) scheme for the detection and localization of cracks in steel rail under loads. In the operation, AE signals were captured by the AE sensor and converted into digital signal data by AE data acquisition module. The digital data were denoised to remove ambient and wheel/rail contact noises, and the denoised data were processed and classified to localize cracks in the steel rail using a deep learning algorithmic model. The AE signals of pencil lead break at the head, web, and foot of steel rail were used to train and test the algorithmic model. In training and testing the algorithm, the AE signals were divided into two groupings (150 and 300 AE signals) and the classification accuracy compared. The deep learning-based AE scheme was also implemented onsite to detect cracks in the steel rail. The total accuracy (average F1 score) under the first and second groupings were 86.6% and 96.6%, and that of the onsite experiment was 77.33%. The novelty of this research lies in the use of a single AE sensor and AE signal-based deep learning algorithm to efficiently detect and localize cracks in the steel rail, unlike existing AE crack-localization technology that relies on two or more sensors and human interpretation.

## 1. Introduction

Rail transport plays an important role in transferring a large number of passengers and freight between destinations. The significance of the railway thus necessitates regular structural heath monitoring (SHM) to identify welding discontinuity defects in the rail track and maintain the health of steel rail. Scheduled SHM prevents the incidence of rail accidents and improves passenger comfort.

Nondestructive acoustic emission (AE) testing is a passive technique to detect elastic energy spontaneously released by material with developing cracks [1,2,3,4]. The AE technique is also deployed to characterize the crack behavior under a load. AE is a transient elastic wave in the frequency range of 95 kHz–300 kHz. The advantages of a passive AE technique over conventional active nondestructive testing (NDT) methods include online monitoring, high sensitivity, early and rapid defect detection [5,6,7,8].

The AE signal from cracks in the steel rail is caused by fatigue in wheel/rail contact. If improperly attended to, the rail crack induced by dynamic wheel/rail contact and interaction could result in disastrous failures [9,10,11,12,13]. The defects in steel rail are predominantly fatigue cracks [14,15,16].

The conventional AE technology (without machine learning) has been applied to inspection and monitoring of building or structural integrity, pressure vessel, pipeline, aircraft components, storage tanks, tanks on tank ship and in material research [17,18,19,20]. In structural integrity, AE testing was used to inspect and monitor emerging fatigue cracks in in-service steel bridges [21,22,23]. In a pressure vessel, the AE technology was used for online monitoring of fatigue cracks in liquified petroleum gas (LPG) and natural gas for vehicle (NGV) pressure vessels under a load [24,25]. AE was also used to detect and locate corrosion and leakage in underground oil, LPG, and NGV pipelines [26,27].

In aircraft components, the AE technology was used to detect fatigue cracks under a load [28,29,30]. In a storage tank, AE testing was used to monitor the storage tank health and to detect and locate corrosion and leakage in the bottom plate of storage tank [31,32]. In material research, the AE technology was used to characterize material properties, failures, and crack mechanics [33,34]. In a railway, the technology was utilized to detect and localize fatigue cracks in the steel rail and rail components under a load [35,36,37]. However, the conventional AE technology for detection and localization of fatigue cracks relies on two or more sensors and on human interpretation, which is prone to error.

To address the human error inherent in the conventional AE technology, image-based deep learning was integrated into the AE technology to localize crack sources in plate-like structures [38]. In a spherical water storage tank, the AE scheme with support vector machine (SVM) was used to classify corrosion and leakage but the scheme failed to localize the source [39]. In a journal bearing, the AE scheme with SVM was used to detect friction and wear [40]. In substation transformer, the AE technology with SVM was used to detect electrical treeing [41]. In [42], an acoustic sensor array with SVM algorithms was proposed to detect shock and vibration in fan matrices. In composite materials, the AE technology with a nonhomogeneous hidden semi-Markov (semi-probability) model was used to predict the remaining useful life and fatigue [43]. 

Specifically, in [38], a single-sensor AE scheme based on image-based deep learning was proposed to localize defects in plate-like structures. Their proposed scheme utilized a single sensor, similar to this current research. Nevertheless, the deep learning algorithm was image-based while the algorithm of this research is signal-based. In [39,40,41,42], multi-sensor and sensor array AE schemes with SVM algorithms were used to detect defects in material. Unlike this current research, their schemes utilized two or more sensors or sensor-array and SVM algorithms. In [43], a multi-sensor AE scheme with hidden semi-Markov (semi-probability) model was proposed for in-situ prognostics of fatigue life of composites. Although the image-based deep learning could localize crack sources, the image-based algorithm requires more computational resources than the proposed AE signal-based classification deep-learning algorithmic scheme. 

As a result, this research proposes a nondestructive single-sensor AE scheme with AE signal-based deep learning algorithm for detection and localization of cracks in the steel rail under a load. The AE signals of pencil lead break (PLB) at the head, web, and foot of steel rail were used to train and test the deep learning algorithmic model. In training and testing the algorithm, the AE signals were divided into two groupings and the classification accuracy compared. The AE scheme was subsequently implemented onsite to detect cracks in the steel rail and a comparison of the resulting classification performance was conducted. 

## 2. The Proposed Single-Sensor AE Scheme

**Figure 1:**
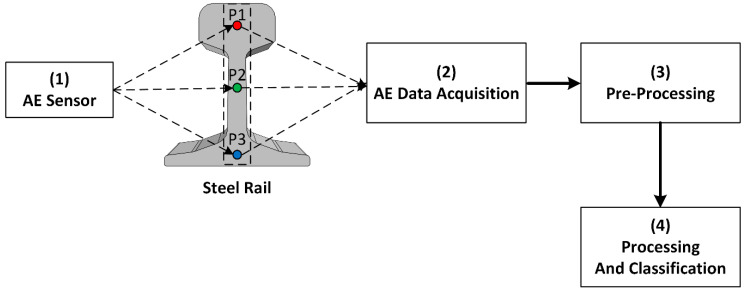
The diagram of the nondestructive single-sensor acoustic emission (AE) scheme for crack detection in steel rail.

Figure 1 illustrates the diagram of the proposed nondestructive single-sensor AE scheme for crack detection in steel rail. Under the proposed scheme, AE signals were captured by the AE sensor and converted into digital signal data by AE data acquisition module. The digital signal data were pre-processed to remove noises using a total variation denoising algorithm. The denoised digital signal data were processed and classified to localize cracks in the steel rail. In the figure, P1, P2, and P3 denote rail head, rail web, and rail foot, respectively. 

### 2.1. Acoustic Emission Sensor 

Acoustic emission (AE) is the transient elastic waves in solids that occur when the internal structure of a material undergoes deformation due to plastic deformation, crack propagation, erosion, corrosion impact, leakage, and fatigue. This research focused on the AE signals caused by fatigue cracks in the steel rail under a load.

The AE sensor was Vallen model VS150-RIC with an 80 kHz–500 kHz bandwidth and −40 °C to +85 °C operating temperatures. Given the natural frequency of cracks at 150 kHz, the AE sensor was used to capture crack signals (elastic waves) in the steel rail. The AE sensor was of piezoelectric type to convert elastic waves in the steel rail into electrical AE signal. To mitigate the triboelectric effect, the AE sensor of the proposed scheme was integrated electronics piezoelectric (IEPE) sensor, as opposed to the non-IEPE sensor which is more prone to the triboelectric effect. To further mitigate the triboelectric effect, the bandpass filter frequency range of the proposed deep learning-based AE sensor scheme was set at 95 kHz–300 kHz.

### 2.2. AE Data Acquisition Module

The AE signals captured by the AE sensor were converted into digital signal data by an AE data acquisition module (Vallen AMSY-6) with 16-bit analog-to-digital conversion (ADC). The sampling rate of ADC conversion was 20 MHz. A computer was used for storage of digital signal data.

### 2.3. Pre-processing of AE Signal Data

**Figure 2:**
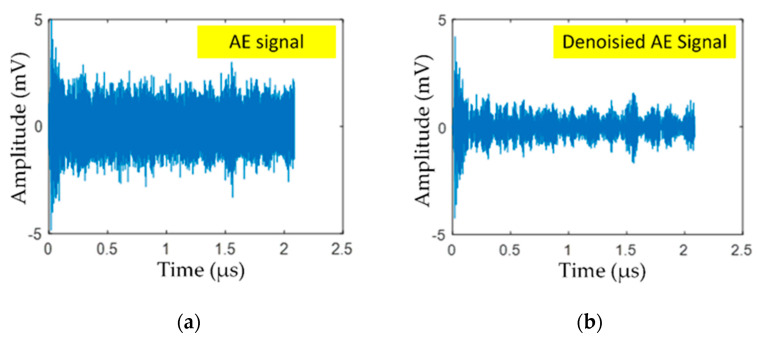
The AE signal of cracks in steel rail: (**a**) AE signal, (**b**) denoised AE signal using total variation denoising (TVD).

Total variation denoising (TVD) algorithm was used to remove noise in the AE signal of cracks in a steel rail. In practical use, TVD is effective in removing ambient noise and rolling noise. Figure 2a shows the AE signal of cracks in steel rail with ambient and wheel/rail noise, and Figure 2b illustrates the denoised AE signal using TVD.

The AE signal with ambient noise and rolling noise is mathematically expressed in Equation (1).

(1) y=x+w

where *y* is noisy AE signal, x is unnoisy AE signal, and *w* is additive white Gaussian noise. 

Given that x is a piecewise constant signal with sparse representation in first-order derivative, the estimated AE signal ($\hat{x}$) can be solved by minimizing the cost function (i.e., the first term on the right-hand side; *F*(x)), as expressed in Equation (2).

(2) $$\hat{x}=\underset{x}{\mathrm{argmin}}\frac{1}{2}{||y-x||}_{2}^{2}+\lambda {||Dx||}_{1}$$

where *λ* is the regularization parameter and *D* is the first-order difference operator, as expressed in Equation (3).

(3) $$D=\left[\begin{array}{ccc} \begin{array}{c} -1 \end{array} & \begin{array}{c} 1\\ -1 \end{array} & \begin{array}{cc} \begin{array}{c} 1\\ \begin{array}{c} \ddots \end{array} \end{array} & \begin{array}{cc} \begin{array}{c} \begin{array}{c} \ddots \\ -1 \end{array} \end{array} & \begin{array}{c} \begin{array}{c} 1 \end{array} \end{array} \end{array} \end{array} \end{array}\right]$$

**Figure 3:**
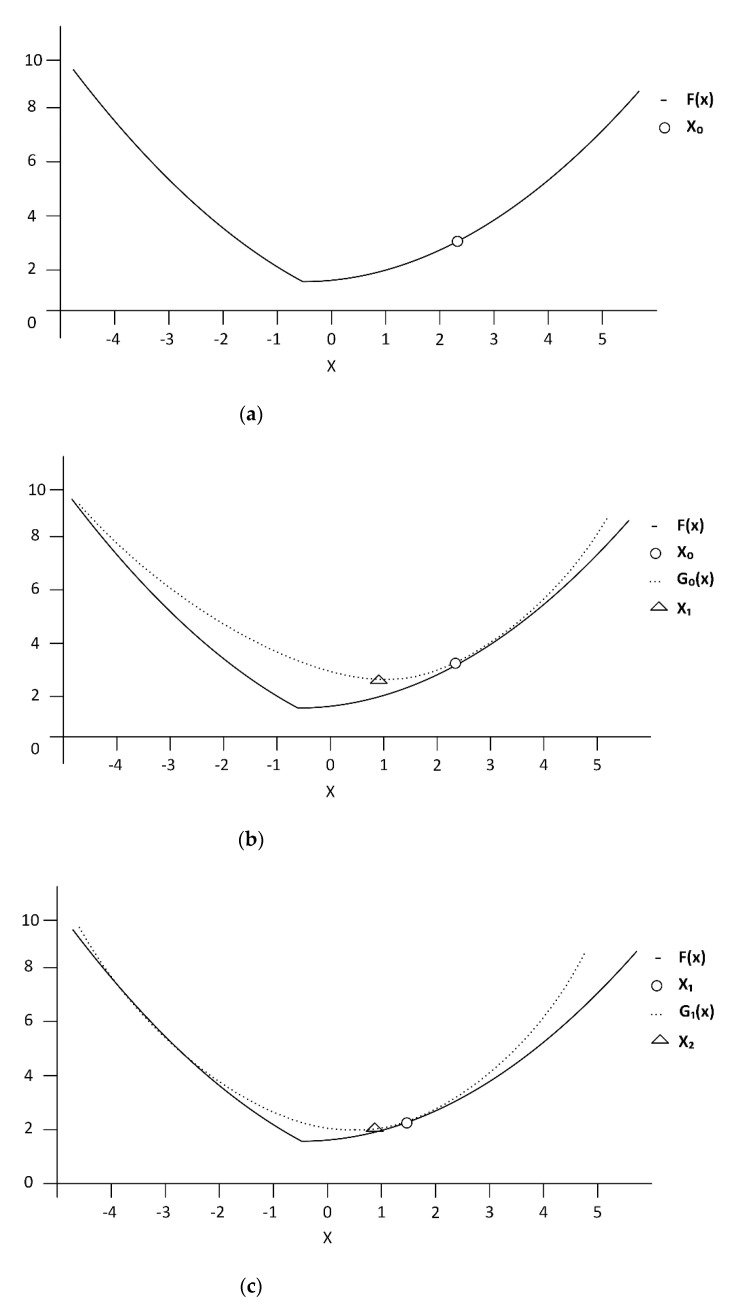
Majorization-minimization (MM) procedure: (**a**) cost function *F*(x) to be minimized and initial x*_o_*; (**b**) iteration 1 where majorizer *G*_0_(x) is tangent to *F*(x) at x_0_ and minimize *G*_0_(x) for x_1_; (**c**) iteration 1 where majorizer *G*_1_(x) is tangent to *F*(x) at x_1_ and minimize *G*_1_(x) for x_2_.

The estimated AE signal ($\hat{x}$) (Equation (2)) could also be efficiently solved by majorization-minimization (MM) algorithm [32,33]. Rather than direct minimization of the cost function (*F*(x)), MM algorithm solves a sequence of optimization problems (*G_k_*(x), where *k* = 0, 1, 2,…), based on the premise that solving for *G_k_*(x) is less demanding than solving for *F*(x). MM generates a sequence of x*_k_*, each derived by minimizing *G_k_*_−1_(x); and MM algorithm iterates until arriving at minimum *F*(x), as shown in Figure 3. 

In the MM algorithm, the functions *G_k_*(x) should be convex functions and must be specified such that it closely approximates the cost function (*F*(x)). In addition, MM algorithm requires that individual *G_k_*(x) function be a majorizer of *F*(x) (i.e., *G_k_*(x) ≥ *F*(x), ∀x) and that the *G_k_*(x) function is tangent to *F*(x) function at x*_k_* (i.e., *G_k_*(x*_k_*) = *F*(x*_k_*)). In this research, the parabolic functions *G_k_*(x) were used to approximate the minimum *F*(x), which is the optimal denoised AE signal ($\hat{x}$) as shown in the Algorithm 1.

**Algorithm1:** The MM approach to minimize the cost function (*F*(x)) (i.e., the optimal denoised AE signal ($\hat{x}$) can be summarized as follows [44]:
(1)Set *k* = 0, and initialize x_0_.(2)Choose *G_k_*(x) such that *G_k_*(x) ≥ *F*(x) for all x and *G_k_*(x*_k_*) = *F*(x_*k*_)(3)Set x_*k*+1_ as the minimizer of *G_k_*(x), as shown in Equation (4).(4)$x_{k+1}=\;\arg\underset{x}{\min}G_{k}\left(x\right)\;$(4)Set *k* = *k* + 1 and repeat step (2) and iterate until arriving at the minimum *F*(x).


### 2.4. Processing and Classification

**Figure 4:**
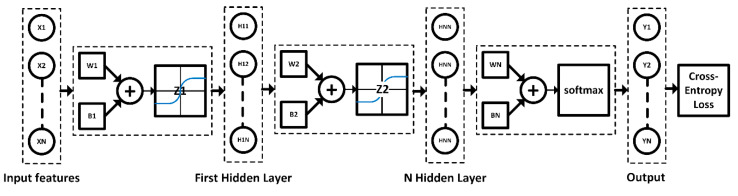
The typical deep learning algorithm for classification.

Figure 4 illustrates the typical deep learning algorithm for classification, consisting of the input (feature) layer, hidden layers through to *N* hidden layer, and output (target) layer. In this research, the activation function between hidden layers was hyperbolic tangent function (*tanh(z)*) since the function is ideal for the transient nature of AE signal and is capable of suppressing noises and interferences. The softmax activation function (*softmax(z)*) was used for classification based on probability. The aim of deep learning algorithm for classification is to localize cracks in the rail head, rail web, and rail foot of steel rail. 

Prior to algorithm training and validation, input (feature) data were scaled using standardization to normalize, as shown in Equation (5).

(5) $$Standardization\;=\frac{X-Mean\;X}{SD}$$

where *x* is the feature (input data), Mean *x* is the mean value of feature, and *SD* is standard deviation.

In training the algorithmic model, the initial weight (*W*) and bias (*B*) (i.e.,$W_{1},\;B_{1,}W_{2},\;B_{2},\;W_{3},\;B_{3},W_{4},B_{4}$), were random. In feedforward, hyperbolic tangent function (*tanh(z)*) was the activation function between hidden layers, as shown in Equation (6) where *tanh(z) =* [−1,1]. The output layer used *softmax(z)* as the activation function for classification based on probability, as shown in Equation (7) and the linear combination, as shown in Equation (8).

(6) $$tanh\left(z\right)=\frac{\left(e^{z}-e^{-z}\right)}{\left(e^{z}+e^{-z}\right)}$$

(7) $$softmax\left(z\right)=\frac{e^{Z_{i}}}{\sum_{j=1}^{k}e^{z_{j}}}$$

(8) $$Z=\left[\begin{array}{c} z_{1}\\ z_{2}\\ \begin{array}{c} \vdots \\ z_{N} \end{array} \end{array}\right]=\left[\begin{array}{ccc} \begin{array}{c} x_{1}^{1}w_{1}\\ x_{2}^{1}w_{1}\\ \begin{array}{c} \vdots \\ x_{N}^{1}w_{1} \end{array} \end{array} & \begin{array}{c} x_{1}^{2}w_{2}\\ x_{2}^{2}w_{2}\\ \begin{array}{c} \vdots \\ x_{N}^{2}w_{2} \end{array} \end{array} & \begin{array}{cc} \begin{array}{c} \ldots \\ \ldots \\ \begin{array}{c} \vdots \\ \ldots \end{array} \end{array} & \begin{array}{c} x_{1}^{D}w_{D}\\ x_{2}^{D}w_{D}\\ \begin{array}{c} \vdots \\ x_{N}^{D}w_{D} \end{array} \end{array} \end{array} \end{array}\right]+\left[\begin{array}{ccc} B_{1} & B_{2} & \begin{array}{cc} \ldots & B_{D} \end{array} \end{array}\right]$$

Partial derivative of the hyperbolic tangent function (*tanh(z)*) was used in back propagation, as shown in Equation (9).

(9) $$\frac{\partial \left[tanh\left(z\right)\right]}{\partial z}=1-{tanh}^{2}\left(z\right)={\mathrm{sech}}^{2}\left(z\right)$$

The cross-entropy loss function for multiclass was used to fine-tune the optimization of the algorithmic model, as shown in Equation (10).

(10) $$J\left(w\right)=-\frac{1}{N}\sum_{n=1}^{N}(Y_{n}\log({\hat{Y}}_{n}))$$

where $Y_{n}$ is true output value and ${\hat{Y}}_{n}$ is predicted output value.

## 3. Experimental Material and Setup

### 3.1. Experimental Steel Rail

The experimental steel rail was of UIC 54 type, which is the steel rail commonly used in countries in the Southeast Asian region. The elemental composition of UIC 54 steel rail was analyzed by handheld laser-induced breakdown spectroscopy (LIBS) analyzer (SciAps, model Z-300). Table 1 tabulates the elemental composition of UIC 54 steel rail.

### 3.2. AE Data Sensor and Acquisition Module

**Figure 5:**
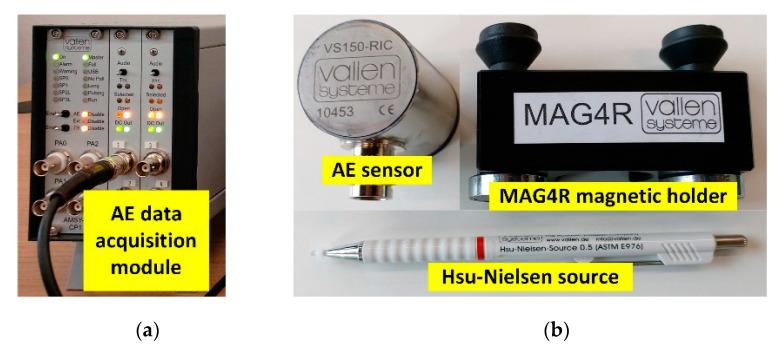
AE data sensor and acquisition: (**a**) AE data acquisition module; (**b**) AE sensor, MAG4R magnetic holder, and Hsu–Nielsen source.

Figure 5a shows the AE data acquisition module with 16-bit ADC and a sampling rate of 20 MHz (Vallen AMSY-6). Figure 5b illustrates the AE sensor (S/N 10453) with 80 kHz–500 kHz bandwidth and −40 °C to +85 °C operating temperatures (Vallen model VS150-RIC). The MAG4R magnetic holder was used to attach the AE sensor to the steel rail (on the field side of the rail head).

The Hsu–Nielsen source was used as the artificial source of AE signal of crack, consisting of 2H pencil lead (0.5 mm), guide tube, and mechanical pencil. The pencil lead was broken against the head, web, and foot of steel rail at 30°, in accordance with the American Society for Testing and Materials (ASTM E976) standard. 

### 3.3. AE Sensor Amplitude Testing

The AE sensor amplitude was analyzed prior to capturing the AE signals of cracks in the steel rail to determine the sensor sensitivity and sensor coupling. The AE sensor amplitude testing was carried out at the rail head by breaking 2H pencil lead (0.5 mm) against the steel rail. The testing was performed five consecutive times and results averaged. 

**Figure 6:**
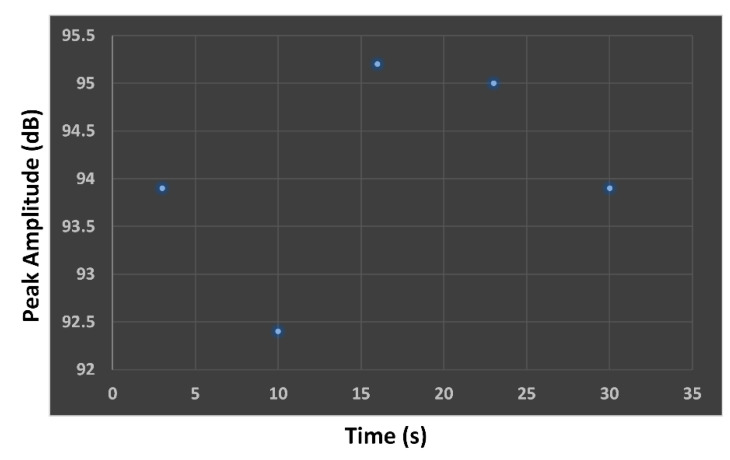
Peak amplitude of AE sensor relative to time.

Figure 6 illustrates the peak amplitude (dB) of the AE sensor relative to time (s), and the results are tabulated in Table 2. In Figure 6, the maximum peak amplitude of the experimental AE sensor was greater than 80 dB (>80 dB), indicating that the AE sensor could be deployed to capture the AE signals of cracks in the steel rail.

### 3.4. Datasets for Training and Testing the Deep Learning Algorithmic Model

**Figure 7:**
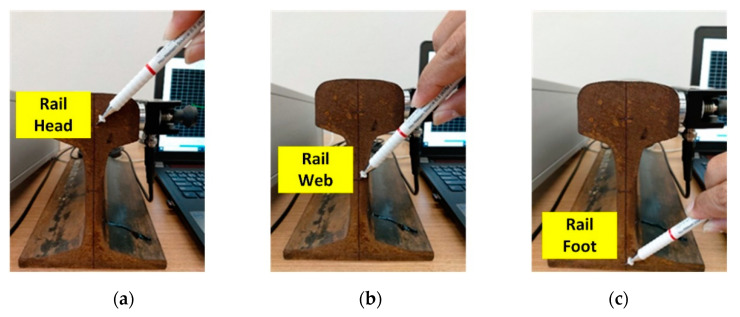
Locations of pencil lead break (PLB): (**a**) rail head, (**b**) rail web, (**c**) rail foot.

The digital signal data of PLB at the head, web, and foot of steel rail were datasets for training and testing the proposed deep learning algorithmic model. PLB were carried out 150 times each at the head, web, and foot of steel rail; and the AE signals were captured, totaling 450 AE signals (Figure 7). 

The un-denoised AE signals were subsequently divided into two groupings (150 and 300 AE signals) to investigate the effect of number of input data on the classification accuracy of the deep learning algorithmic model. Under the first grouping (150 AE signals), the input data were divided into a training dataset (80% of the input data) and a testing dataset (20%). Under the second grouping (300 AE signals), 80% of the input data were training dataset and the rest (20%) were testing dataset. 

**Figure 8:**
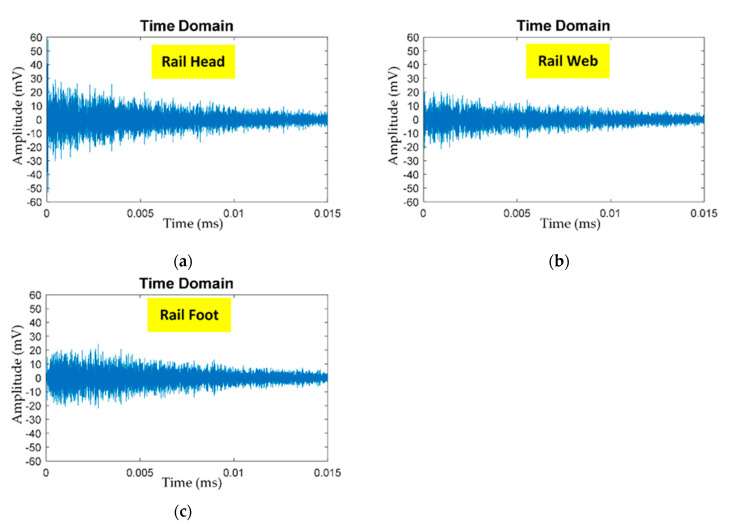
Un-denoised AE signals of PLB at: (**a**) rail head, (**b**) rail web, (**c**) rail foot.

Figure 8a–c shows the un-denoised AE signals (prior to pre-processing) of PLB at the head, web, and foot of steel rail. The AE signals of PLB were captured by the AE sensor and converted by the AE acquisition module into un-denoised AE digital signal data prior to pre-processing by total variation denoising (TVD) algorithm to remove the ambient noise.

**Figure 9:**
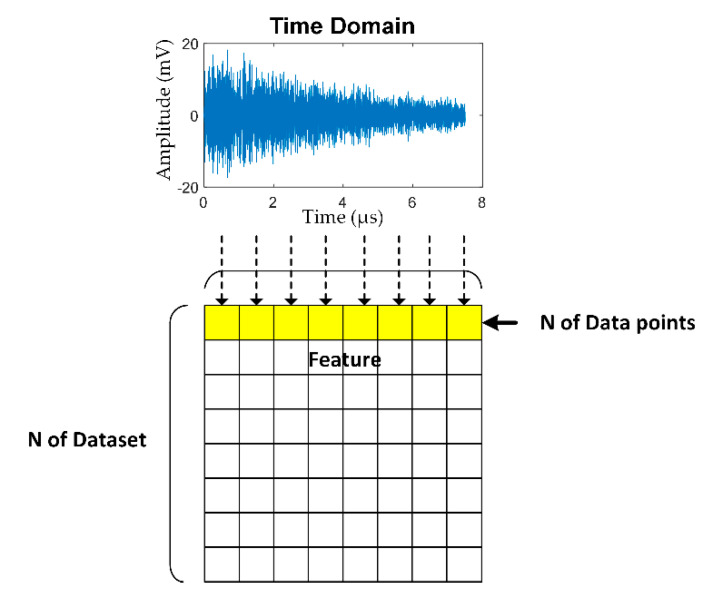
Conversion of denoised AE signals (after pre-processing) of PLB into dataset for training and testing the deep learning algorithmic model.

Figure 9 illustrates the conversion procedure of denoised AE signals (after pre-processing) of PLB into feature datasets (input data) for training and testing the deep learning algorithmic model. The training and testing datasets were transferred to a spreadsheet. The feature datasets were divided into two groupings (150 and 300 feature datasets) to investigate the effect of number of input data on the classification accuracy of the deep learning algorithmic model. 

Under the first grouping (150 feature datasets), 50 datasets each belonged to PLB at the head, web, and foot of steel rail. The feature datasets were divided into training dataset (80%) and testing dataset (20%). Under the second grouping (300 feature datasets), 100 datasets each belonged to PLB at the head, web, and foot of steel rail. Likewise, 80% of the input data were training dataset and the rest (20%) were testing dataset. Given the sampling rate of 20 MHz of the AE acquisition module, one feature dataset (i.e., one denoised AE signal) contained 300,000 data points. 

In this research, one AE signal contains 300,000 data points, and the time to capture one data point is 0.05 µs, given f_s_ = 20 MHz. As a result, the time required to capture one AE signal (i.e., 300,000 data points) is 0.015 s (= 300,000 × 0.05 × 10^−6^).

**Figure 10:**
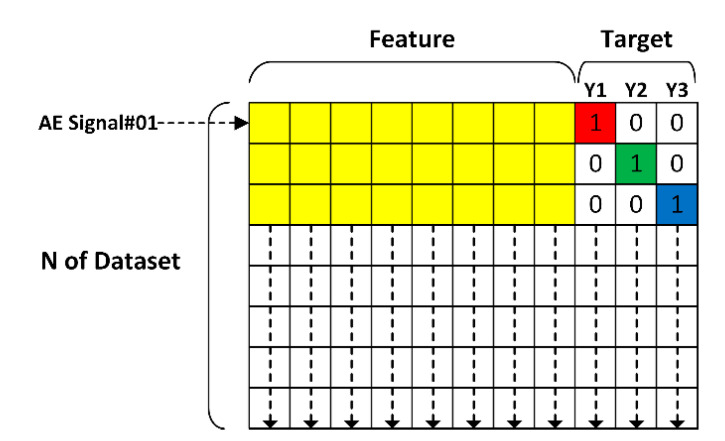
Feature and target datasets for training and testing the deep learning algorithmic model.

In Figure 10, one row of yellow grid cells represents one AE signal (300,000 data points), and the total number of rows (i.e., N of Dataset) represents 450 AE signals (PLB digital signal data = 450 signals) at the rail head, web, and foot. In the columns “Target”, the rail head, rail web, and rail foot are represented by red, green, and blue colors, respectively. 

In training the deep learning algorithm, a specific target (either rail head, web, or foot) was assigned to each row of yellow grid cells (i.e., each AE signal) using one-hot encoding, where 1 denotes a 100% probability and 0 a zero probability. The training was carried out separately for the first grouping (80% of the input data = 120 signals) and second grouping (80% of the input data = 240 signals) of AE signals.

In testing the deep learning algorithm, a given AE signal was applied to the trained deep-learning algorithm and the algorithm classified the crack location based on probability, where Y1, Y2, and Y3 denote the head, web, and foot of steel rail, respectively. In testing the algorithm, there were 30 signals (20% of the input data) for the first grouping, consisting of 10 signals each for rail head, web, and foot. Meanwhile, there were 60 signals (20% of the input data) for the second grouping, consisting of 20 signals each for rail head, web, and foot. 

The following example is to show the testing process of the algorithm, assuming that the deep learning-generated probability of Y1, Y2, and Y3 of the first row of yellow grid cells (AE signal#01) are 0.1 (10%), 0.4 (40%), and 0.5 (50%), the proposed deep learning algorithm would select Y3 (rail foot) as the location of the crack, based on the highest probability value. 

### 3.5. Proposed Deep Learning Algorithm for Classification

**Figure 11:**
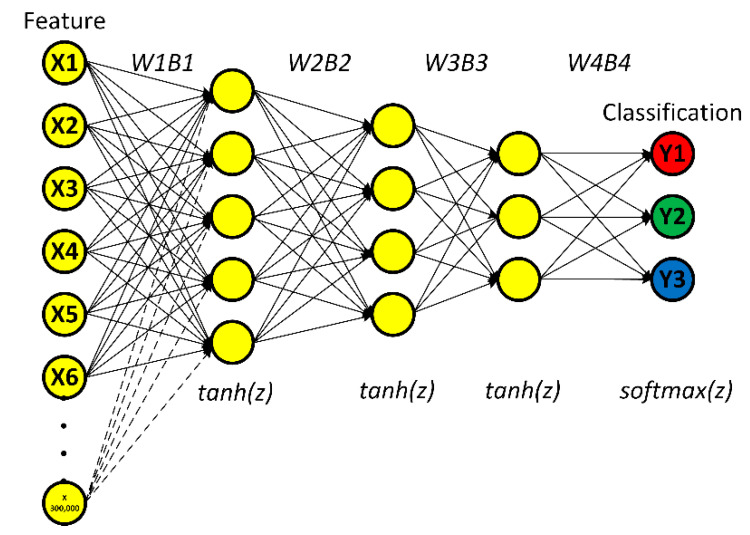
The proposed deep learning algorithm for classification.

Figure 11 illustrates the proposed deep learning algorithm for classification. The AE data points were input of the feature layer, and one AE signal contained 300,000 data points, as represented by X1, X2, X3…X300,000. In this research, there were 450 AE signals of PLB under two groupings.

The proposed algorithmic model consisted of three hidden layers and one output layer. The first, second, and third hidden layers had 5, 4, and 3 nodes, respectively. The activation function between hidden layers was hyperbolic tangent function (*tanh(z)*), which transforms linear to nonlinear function. The output layer consisted of Y1 (crack in the rail head), Y2 (crack in the rail web), and Y3 (crack in the rail foot). The softmax activation function (*softmax(z)*) was used for classification based on probability. The aim of the proposed deep learning algorithm for classification is to localize cracks in the rail head, rail web, and rail foot of steel rail.

**Figure 12:**
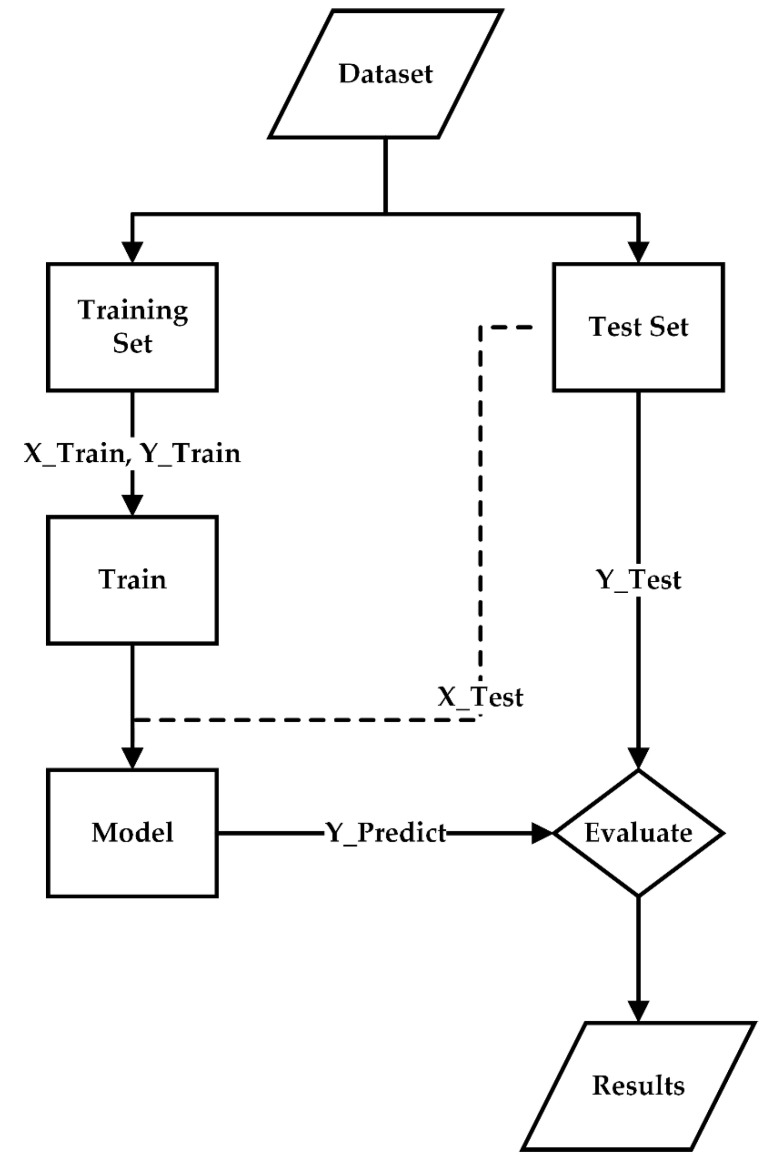
Training and testing the proposed deep learning algorithm and the model validation.

Figure 12 illustrates the process of training and testing the proposed deep learning algorithmic model and the model validation. The AE datasets (feature and target datasets) were divided into a training dataset (80%) and a testing dataset (20%).

The training dataset consisted of X_Train (normalized feature/input dataset) and Y_Train (target/output dataset), while the testing dataset comprised X_Test (normalized feature dataset) and Y_Test (target dataset). The initial weight (*W*) and bias (*B*) in the hidden layers ($W_{1},\;B_{1,}W_{2},\;B_{2},\;W_{3},\;B_{3},W_{4},B_{4}$) were random, given the learning rate (α) of 0.1 and epoch of 1000. *W* and *B* were fine-tuned by gradient descent iterative optimization algorithm. 

In the training and testing processes, L1-norm regularization was utilized to avoid overfitting due to excessive data points (300,000 data points per AE signal) and divergence between the cross-entropy loss of training and testing datasets. 

The classification results of the optimized algorithmic model (Y_Predict) were compared against the training (Y_Train) and testing target (Y_Test) datasets to evaluate the classification performance of the proposed deep learning algorithm.

### 3.6. Evaluation of the Deep Learning Algorithm

The classification performance of the proposed deep learning algorithmic model was evaluated by comparing the classification results using the algorithmic model against the training and testing target datasets. The performance metrics were the receiver operating characteristic (ROC) curve, confusion matrix, F1 score, and total accuracy (average F1 score). 

#### 3.6.1. Receiver Operating Characteristic (ROC) Curve 

A receiver operating characteristic (ROC) curve shows the classification performance of an algorithmic model whereby the area under the ROC curve is used as a performance indicator of the model. The ROC curve plots two parameters: true positive rate (y-axis) and false positive rate (x-axis). The true positive rate (TPR) and false positive rate (FPR) can be calculated by Equations (11) and (12), respectively.

(11) $$True\;positive\;rate\;\left(TPR\right)\;=\;\frac{TP}{TP+FN}$$

where *TP* is the number of true positives classified by the algorithmic model and *FN* is the number of false negatives classified by the algorithmic model.

(12) $$False\;positive\;rate\;\left(FPR\right)=\;\frac{FP}{TN+FP}$$

where *FP* is the number of false positives classified by the algorithmic model and *TN* is the number of true negatives classified by the algorithmic model. 

#### 3.6.2. Confusion Matrix, F1 Score and Total Accuracy (Average F1 Score)

The confusion matrix is a table layout that enables visualization of the classification performance of an algorithmic model. F1 score is a value that indicates the classification performance of the algorithmic model using Precision (Equation (13)) and Recall. The Recall equation in the F1 Score calculation is identical to Equation (11) (true positive rate). As a result, equation (11) can be used in the Recall calculation. F1 Score, Individual-class Accuracy and Total accuracy (the average of F1 scores) can be calculated by Equations (14)–(16), respectively.

(13) $$Precision\;=\;\frac{TP}{TP+FP}$$

(14) $$F1\;\mathrm{Score}=\frac{2*Precision*Recall}{Precision+Recall}$$

(15) $$Individual-\mathrm{class}\;\mathrm{Accuracy}=\;\frac{TP+TN}{TP+TN+FP+FN}$$

(16) $$\mathrm{Total}\;\mathrm{Accuracy}\;\left(\;\mathrm{average}\;F1\;\mathrm{Score}\right)=\frac{(F1\;\mathrm{score}\;\left(\mathrm{class}\;0\right)+\;(F1\;\mathrm{score}\;\left(\mathrm{class}\;1\right)+(F1\;\mathrm{score}\;\left(\mathrm{class}\;2\right)}{3}$$

## 4. Onsite Experimental Setup

### 4.1. Onsite Advanced Phased Array Ultrasonic Testing (PAUT) 

**Figure 13:**
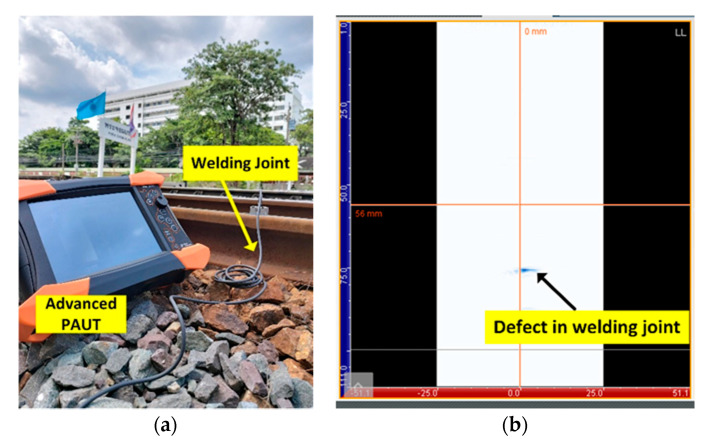
Onsite experimental setup: (**a**) advanced phased array ultrasonic testing (PAUT) on welding joint of the steel rail; (**b**) PAUT detection with defect in the welding joint.

Prior to the implementation of the proposed AE scheme for crack detection in steel rail, advanced PAUT with 5 MHz 64-channel linear array probe (model GEKKO; M2M, France) was applied to welding joints of the steel rail to determine and localize cracks. The PAUT detection was carried out on the welding joints due to their susceptibility to subsurface crack. The proposed AE scheme was subsequently applied to the same welding joints with defects, and the detection results of the AE scheme were validated against those of PAUT to evaluate the classification performance of the deep learning algorithmic model. Figure 13a,b show the advanced PAUT on a welding joint of the steel rail and the defect in the welding joint.

### 4.2. Steel Rail Temperatures

**Figure 14:**
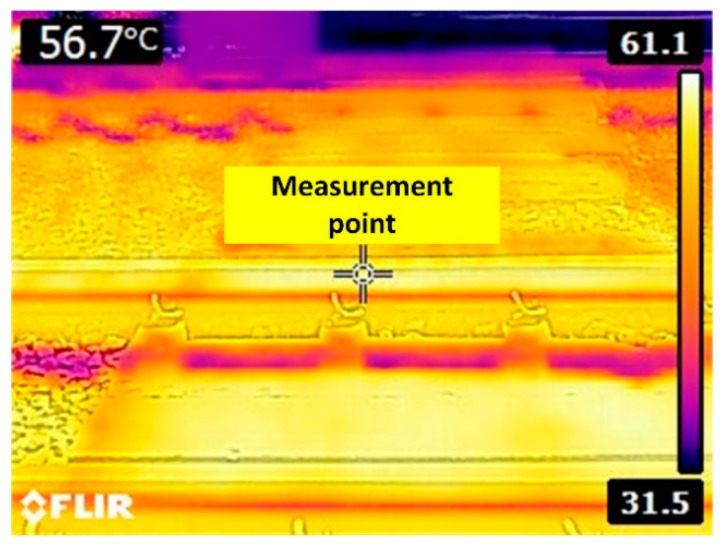
Steel rail temperature measurement.

Given the operating temperature range of −40 to 85 °C of the experimental AE sensor, the temperatures of steel rail around mid-day were measured by using a thermal imaging camera (FLIR, USA.) prior to the installation of the AE sensor. The steel rail temperatures were 56.7–61.1 °C, as shown in Figure 14. The steel rail temperatures were within the operating temperature range of the AE sensor.

### 4.3. Onsite Experiment on the Steel Rail under Load

**Figure 15:**
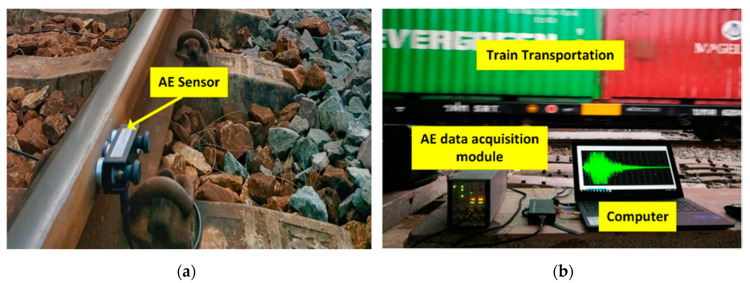
Onsite experiment on steel rail using the AE scheme: (**a**) installation of the AE sensor on the steel rail; (**b**) AE signal data of the steel rail under a load.

Figure 15a illustrates the installation of the AE sensor on the field side of the head of steel rail to detect crack. Figure 15b shows the onsite experiment of the AE scheme with the steel rail under a load. The AE signals of the steel rail under a load were fatigue cracks induced by wheel/rail contact. The AE signals were captured by the sensor and converted into digital signal data by the data acquisition module. The digital signal data were pre-processed to denoise and processed by the deep learning algorithmic model to localize cracks in the steel rail.

## 5. Results and Discussion

### 5.1. Receiver Operating Characteristic (ROC) Curve

**Figure 16:**
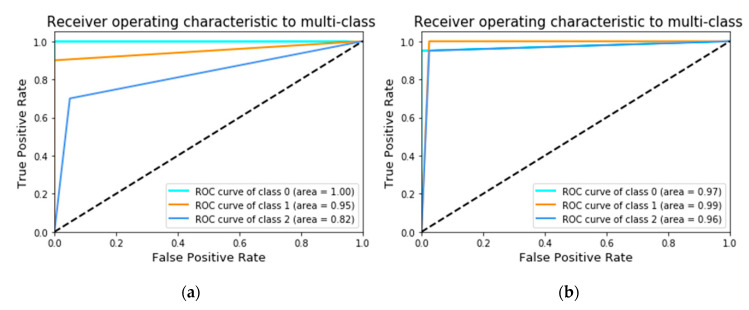
Receiver operating characteristic (ROC) curve where classes 0, 1, and 2 denote rail head, rail web, and rail foot: (**a**) 150 AE signals (first grouping); (**b**) 300 AE signals (second grouping).

The effects of different number of feature datasets (input data) on the classification accuracy of the deep learning algorithmic model were determined by dividing the datasets into two groupings (150 and 300 AE signals). Figure 16a,b respectively illustrate the ROC curves under the first and second groupings, where classes 0, 1, and 2 denote the head, web, and foot of steel rail. 

Under the first grouping, the accuracy performance of the deep learning algorithmic model for class 0 (crack from the rail head), class 1 (rail web), and class 2 (rail foot) were 100% (ROC curve area = 1.0), 95% (0.95), and 82% (0.82). Under the second grouping, the accuracy performance for class 0, class 1, and class 2 were 97% (0.97), 99% (0.99), and 96% (0.96), respectively. The results indicated that the classification accuracy of the deep learning algorithmic model increased with increase in the datasets.

### 5.2. Confusion Matrix of the Deep Learning Algorithmic Model 

**Figure 17:**
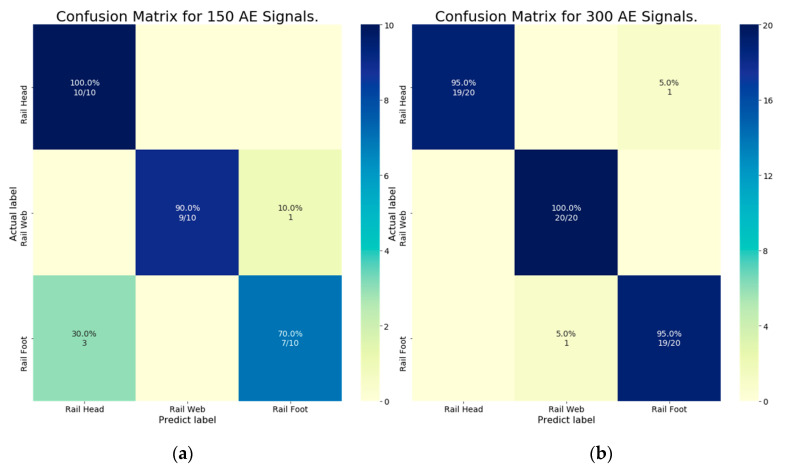
Confusion matrix of the deep learning algorithm for classification: (**a**) 150 AE signals (first grouping); (**b**) 300 AE signals (second grouping).

Figure 17a,b show the confusion matrix of the deep learning algorithmic model for classification under first (150 datasets) and second (300 datasets) groupings of AE signal datasets. In Figure 17a (the first grouping), given 10 crack signals from the rail head (actual), the algorithmic model accurately classified 10 signals (predicted) as from the rail head. For the rail web, out of 10 crack signals (actual), the algorithmic model accurately classified 9 signals (predicted) as from the rail web and erroneously one signal as from the rail foot. For the rail foot, out of 10 crack signals (actual), the model accurately classified 7 signals (predicted) as from the rail foot and erroneously three signals as from the rail head.

In Figure 17b (the second grouping), given 20 crack signals from rail head (actual), the algorithmic model accurately classified 19 signals (predicted) as from the rail head and erroneously one signal as from the rail foot. For the rail web, out of 20 crack signals (actual), the algorithmic model accurately classified 20 signals (predicted) as from the rail web. For the rail foot, out of 20 crack signals (actual), the model accurately classified 19 signals (predicted) as from the rail foot and erroneously one signal as from the rail web.

### 5.3. F1 Scores of the Deep Learning Algorithmic Model 

Table 3 compares the F1 scores and total accuracy of the first (150 AE signals) and second (300 AE signals) groupings. *Precision* and *Recall* were calculated from the confusion matrix (Figure 17). Under the first grouping, the F1 scores for the head, web, and foot of steel rail were 90%, 90%, and 85%, respectively, with a total accuracy of 88.33%. Under the second grouping, the corresponding F1 scores were 95%, 95%, and 92.5%, with a total accuracy of 94.5%. The F1 scores and total accuracy improved as the number of AE signals increased from 150 to 300 signals.

### 5.4. Onsite AE Signals Using the Proposed AE Scheme 

**Figure 18:**
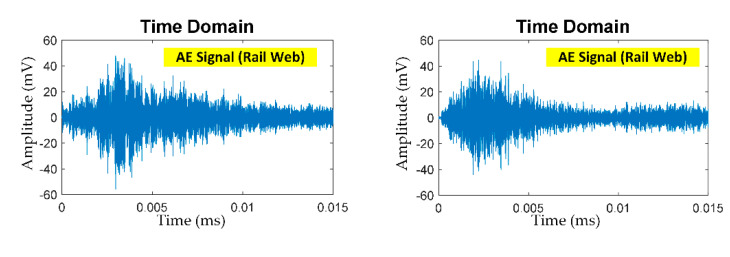
AE signals of cracks in the welding joint of the steel rail under a load using the single-sensor AE scheme.

Figure 18 shows the AE signals of cracks in the welding joint of the steel rail under a load using the nondestructive single-sensor AE scheme. Prior to the implementation of the proposed AE sensor scheme under a load, the advanced PAUT was first used to localize cracks in the steel rail, and the crack locations were used as reference (i.e., actual results). The crack localization by PAUT was carried out at welding joints of the steel rail since the welding joints are highly susceptible to crack. In the crack location, the PAUT probe was placed on top of the steel rail above the welding joint (offline monitoring), in accordance with ASTM E2700 standard (Figure 13).

The proposed AE sensor scheme was then implemented under a load (online monitoring) at the same welding joints previously analyzed by the PAUT. The proposed deep learning algorithm localized the crack in steel rail for each AE signal (from steel rail under a load) based on the highest deep learning-generated probability value. The crack localization results by the proposed deep learning-based AE sensor scheme were compared against the reference (actual results by the PAUT). In this research, the F1 scores (classification accuracy) of the proposed AE sensor scheme for onsite localization of cracks in the rail head, web, and foot were 78%, 80%, and 74%, compared with the PAUT results. The total accuracy (average F1 score) of the proposed AE sensor scheme was 77.33%. 

## 6. Conclusions

This research proposed the deep learning-based single-sensor AE scheme for localization of cracks in the head, web, and foot of steel rail under a load. The AE signals captured by the AE sensor were converted into digital signal data and denoised using a TVD algorithm. The denoised signal data were used to localize the cracks by using a deep learning algorithm. The proposed AE sensor scheme was also implemented onsite to detect cracks in the head, web, and foot of steel rail. The experimental results showed that the classification accuracy (F1 scores) of the AE sensor scheme for onsite localization of cracks in the rail head, web, and foot were 78%, 80%, and 74%, compared with the PAUT results. The total accuracy (average F1 score) of the AE sensor scheme was 77.33%. To improve the total accuracy (average F1 score), subsequent research should increase the number of AE signal data in training the deep learning algorithm, in addition to experimenting with alternative pre-processing algorithms with a higher denoising capability.

## Figures and Tables

**Table 1:** Elemental composition of UIC 54 steel rail.

%C	%Si	%Mn	%V	%Ni	%Cu
0.68	0.22	1.18	0.003	0.0068	0.0109

**Table 2:** Peak amplitude and average amplitude of the AE sensor.

Sensor S/N	1st PLB	2nd PLB	3rd PLB	4th PLB	5th PLB	Average
10,453	93.90	92.40	95.20	95.00	93.90	94.08

**Table 3:** Comparison between F1 score and total accuracy of the first (150 AE signals) and second groupings (300 AE signals).

	Rail Head (150 AE Signals)	Rail Head (300 AE Signals)	Rail Web (150 AE Signals)	Rail Web (300 AE Signals)	Rail Foot (150 AE Signals)	Rail Foot (300 AE Signals)
**Precision (%)** (Equation (13))	76.9	100	100	95.2	87.5	95
**Recall (%)** (Equation (11))	100	95	90	100	70	95
**F1 score (%)** (Equation (14))	86.9	97.4	94.7	97.5	77.7	95
**Accuracy (%)** (Equation (15))	90	98	96	98	86	96
**Total Accuracy (%)** (Equation (16))			86.6 (150 AE signals)			
**Total Accuracy (%)** (Equation (16))			96.6 (300 AE signals)			

## Data Availability

Not applicable.
